# Manchester's Misfortune: The Retirement of Mr. Walter G. Carnt

**Published:** 1915-09-04

**Authors:** 


### MANCHESTER'S MISFORTUNE.

### The Retirement of Mr. Walter G. Carnt.

Manchester's experience of hospitals, their working and provision, has been exceptional and remarkable. The present writer is old enough to have been associated with the difficulties and problems presented by the old infirmary, and to remember the bitter and prolonged differences of opinion which resulted from questions arising in connection with its removal to the present site. In those days one of the superintendents was a gentleman of magnetic influence, who lived in style and was provided with a brougham and horses by the hospital authorities. He was a superintendent to be reckoned with and the personal influence he acquired was remarkable. When at length, after decades of years of dispute and discussion, it was determined to erect a new Royal Infirmary on the Stanley Grove site the committee displayed great wisdom in selecting for the post of general superintendent and secretary Mr. W. G. Carnt, who had undergone special training which eminently qualified him for the post.

Mr. Carnt's parents were wise, for they sent their son to sea in his youth, and after he had seen something of the world and been equipped with health he obtained in 1880 a clerkship in His Majesty's Civil Service by competitive examination. He was appointed to the Prisons Department, and for twelve years was attached to His Majesty's Prison at Derby, being subsequently appointed by the Home Secretary clerk to the Visiting Justices of the prison. He was selected on several occasions to proceed to other prisons to take charge of the accounts in times of difficulty, for which services he received the thanks of the Prison Commissioners. During this period he gained valuable experience in building work and the latest systems of drainage.

### Mr. Carnt's First Hospital Experience.

In 1892 the governors of the Derbyshire Royal Infirmary decided to appoint a permanent secretary, and Mr. Carnt was selected from a large number of applicants. Shortly after Mr. Carnt's appointment the rebuilding of this hospital was commenced, and he was actively associated with the building works, with the appeals for the £128,000 needed to pay for them, and with the raising of money for their equipment by means of a bazaar, of which he was the honorary secretary, which realised £4,500 profit. As the buildings neared completion Mr. Carnt became responsible for the furniture and appliances of the new hospital, and most of these were supplied under contracts drawn up by him and were manufactured locally under his immediate supervision. Mr. Carnt impressed his personality and knowledge so strongly upon the Governors that he was soon made superintendent of the hospital. It was a striking fact in the hospital world within the United Kingdom that the Derby Infirmary began to be talked about and to excite widespread and increasing interest about this time. The first buildings were opened in 1894, and were followed by others in 1895, 1896, 1900, and 1906.

### Difficulties Ahead.

It will be noticed that Mr. Carnt from 1880 to 1906—twenty-six years—was continuously engaged upon works which made him necessarily acquainted with the latest developments in institutional and hospital buildings, their planning and hygienic equipment. He further had had a thoroughly practical training in the equipment, furnishing, organisation and administration of a modern hospital which, under his superintendence, attained a high reputation for efficiency. It is essential to emphasise these points at the moment because they demonstrate the difficulties, which will later appear with even greater force, which the committee of the Manchester Royal Infirmary have to face in finding an adequate and competent successor to Mr. Carnt. How many men working to-day in the hospital world are competent, and, if competent, available, to occupy the post of general superintendent at Manchester, with its huge responsibilities and relatively small remuneration of £700, with quarters, coals and light? Manchester men are fond of declaring that what Manchester does to-day the world will do to-morrow. Manchester in this matter, as the facts stand, represents the tail and not the dog of hospital progress. No doubt the applicants may be numerous, but to keep efficient the Manchester Royal Infirmary up to standard the Board require a man of knowledge and ability, whose market value is quite £1,000 a year.

### His Work and Services.

Manchester people were fortunate when in 1906 they secured the services of Mr. Walter G. Carnt as general superintendent and secretary of their Royal Infirmary. This appointment was properly made at the very commencement, for the new buildings had only just begun, and Mr. Carnt's technical knowledge in relation to buildings, equipment, furniture, organisation, and hygienic completeness, together with his organising powers, impressed the board of management, and to their credit secured him the appointment. From his appointment we have had an intimate knowledge and opportunities for close observation of the actual work and services rendered to Manchester by Mr. Carnt. He has always been a man of great energy; so long as he was awake he was nearly always at work, and the work invariably related to the business, progress, and advantage of the Royal Infirmary and its interests. In April 1910, after inspecting the new infirmary, we reported as one result of that inspection (see The Hospital, April 9, 1910, page 54): "The committee showed great wisdom in selecting for the post of superintendent Mr. W. G. Carnt, for the strain and work which devolved upon the general superintendent, the matron, and the lady housekeeper when these new buildings had to be equipped and reduced to order, and due preparations made for the reception of patients, must have been onerous indeed. Work of this kind involves great administrative gifts, great self-denial, and mastery of detail, and an imagination capable of realising exactly how to arrange for the work to be commenced at the right end and systematically carried out, so that the utmost possible economy and expedition may result. All this Mr. Carnt, Miss Sparshott, and Mrs. Morris accomplished quietly and well, so that the patients were transferred, without the slightest hitch and in a few hours, from the old infirmary to the new, where they found everything provided for their comfort and well-being. We have no doubt that the committee, and the residents of Manchester and the district, fully recognise the excellent service thus rendered, and that they appreciate the smooth and efficient working of this vast series of buildings which has been ably brought about by the devotion and ability of these officers."

### An Arduous Responsibility.

No one who has not had to do the work can realise the enormous strain of bearing the whole responsibility of equipping, furnishing, preparing, and opening a new modern hospital. The wear and tear and brain strain, together with the continuous pressure upon the nervous system, of the organising mind and responsible head are as great as the resulting worries of man or woman in the highest position of authority anywhere. Mr. Carnt was made secretary to the Building Committee, and thus became actively associated with the building operations. The equipment and furnishing of the institution was carried out under his direct supervision, all contracts being drawn by him, whilst he was responsible for the bulk of the designs. His experience in raising money for building and maintenance was most valuable to the committee, and the people of Manchester and district subscribed upwards of £100,000 for the Building Fund in less than twelve months so as to free the new hospital from debt before the opening by T.M. King Edward VII. and Queen Alexandra on July 6, 1909.

### Successful Finance and Administration.

For a relatively short period only Mr. Carnt was able to direct his attention to the internal organisation; but additional building operations had to be undertaken, and it soon became necessary to erect a central branch for accidents and out-patients near the site of the old infirmary, and to raise the money to defray its cost. These buildings are completed and occupied by wounded soldiers, but the outbreak of the war, and the many calls upon the public for war funds, interfered with the central branch building fund, which still requires some £2,500 to complete it. We hope this sum will be provided before Mr. Carnt vacates his office. Another most difficult task fell to Mr. Carnt's lot. The new Royal Infirmary, containing so great an increase in the number of beds available, added largely to the yearly expenditure, and the annual income thus became one of the greatest anxieties of the Board of Management. It is still an anxiety, but the annual deficiency is now reaching comparatively reasonable proportions, for, during Mr. Carnt's tenure of office, the receipts, excluding income from investments, have increased from about £9,900 to about £22,900 per annum, a record of which he is justly proud. Within the last twelve months, too, additional equipment, to accommodate 248 wounded soldiers in the infirmary, and 60 at the central branch, has been purchased. Recently under Mr. Carnt's supervision the newly established radium department has been organised for the benefit of the Manchester hospitals, and its equipment was designed and purchased by him.

### Mr. Carnt's Loyalty and Devotion.

Endless, untiring, conscientious, devoted, self-denying effort and the fulfilment of the most active and exacting responsibilities and duties which can fall to a hospital superintendent have for twenty-three continuous years placed a strain upon the constitution and energies of Mr. Carnt, who has been called upon to do the work of two men rather than one during much of this period. No wonder that his constitution has broken down, and in tendering him our sympathy in the disappointment and affliction from which he at present suffers we also tender our sympathy to the committee of the Manchester Royal Infirmary and the people of Manchester, upon whom the grave misfortune of Mr. Carnt's early retirement has fallen. He has rendered yeoman service in full measure with a loyalty and devotion which no acknowledgment can fully repay. Mr. Carnt's work and ability have been recognised through the British Embassy in connection with the Hertford British Hospital in Paris; by H.R.H. Princess Henry of Battenberg in connection with the conversion of the Frank James Memorial Home at East Cowes into a cottage hospital; and by the committee of the Chesterfield General Hospital in connection with extensive building operations there. Amongst hospital workers his fulness of knowledge and ready generosity in helping everyone interested in hospital administration have won for Mr. Carnt the gratitude and recognition of many zealous workers in the voluntary hospital field.

### A Hint to the Wise.

To those who are ambitious of high office in hospital government in this country we commend a study of Mr. Carnt's training and education in hospital work, for it will reveal to them the long and arduous path which must be trodden before efficiency and full knowledge can be adequately acquired. It is no small matter to the voluntary hospitals that a man of Mr. Carnt's ability and knowledge should break down prematurely in his hospital work. Every day the complexity and difficulty of organising, maintaining, and administering our great hospitals tends to increase and develop. It takes years to train a man to make a great hospital superintendent, and at the present time it is difficult to find candidates for training who have the knowledge and education, to say nothing of the spirit and zeal, which are essentials to success. How great the value of Mr. Carnt's continuous supervision and control has been were strikingly illustrated at Derby. We hope that his successor at Manchester, whoever he may be, will prove in practice the very best man that can be found to fill the vacant post.

